# *In vitro* synergistic activity of betulinic acid combined with azoles against pathogenic fungi

**DOI:** 10.3389/fmicb.2026.1835794

**Published:** 2026-06-24

**Authors:** Heng Zhang, Lu Ge, Mengqi Peng, Menghua Tang, Yixin Zeng, Chunmiao Sun, Yi Sun

**Affiliations:** 1 Department of Dermatology, Hubei Provincial Clinical Research Center for Diagnosis and Therapeutics of Pathogenic Fungal Infection, Jingzhou Hospital Affiliated to Yangtze University, Jingzhou, Hubei Province, China; 2 Department of Clinical Medicine, Yangtze University, Jingzhou, Hubei Province, China; 3 Department of Clinical Laboratory, Jingzhou Hospital Affiliated to Yangtze University, Hubei Provincial Clinical Research Center for Diagnosis and Therapeutics of Pathogenic Fungal Infection, Jingzhou, Hubei Province, China

**Keywords:** *Aspergillus*, betulinic acid, *Exophiala dermatitidis*, invasive fungal infections, posaconazole, synergistic effect

## Abstract

**Background:**

Invasive fungal infections and emerging antifungal resistance threaten global public health, demanding effective combination therapies.

**Objective:**

To evaluate *in vitro* synergistic antifungal activity of betulinic acid (BA) combined with four azoles (itraconazole [ITR], voriconazole [VOR], posaconazole [POS], fluconazole [FLC]) against *Aspergillus* spp., *Candida* spp., *Exophiala dermatitidis*, and *Cryptococcus neoformans*.

**Methods:**

Per CLSI M27–A3/M38–A2, broth microdilution checkerboard assay determined BA’s minimum inhibitory concentration (MIC) and synergy with azoles (*n* = 110); flow cytometry measured intracellular reactive oxygen species (ROS) in fungi co–cultured with BA.

**Results:**

BA alone had no antifungal activity, but showed synergy with specific azoles: BA/POS had 82.7% (43/52) synergy against *Aspergillus* spp., 95.2% (20/21) against *E. dermatitidis*, and 70% (7/10) against *Candida auris* (of 28 *Candida* spp., 28.6% synergy). BA/FLC had 55.6% (5/9) synergy against *C. neoformans*. Among the 110 strains, the synergy rates of BA with POS, ITR and VOR were 66.4, 10.9 and 2.7%, respectively; the BA–FLC synergy rate was 18.5% in 27 strains. BA/POS co–culture increased fungal ROS.

**Conclusion:**

BA–POS reduces POS’s MIC and exerts potent synergy against *Aspergillus* spp. and *E. dermatitidis.* BA has potential as an adjuvant for treating *Aspergillus* and *E. dermatitidis* infections.

## 1 Introduction

Fungi are responsible for 1.5 million annual fatalities and have infected one–third of the human population (Dinh et al., 2025; Rodriguez-Leal et al., 2024). These figures exceed the death rates from severe diseases such as malaria, tuberculosis, and breast cancer, and the risk of mortality is particularly high among immunocompromised populations, including patients with hematological malignancies, AIDS, and those who have undergone organ transplantation (Valkovic et al., 2024; Thambugala et al., 2024).

Approximately 90% of invasive fungal infections (IFIs) are caused by the genera *Candida*, *Cryptococcus*, and *Aspergillus*, each presenting distinct therapeutic challenges: Infections caused by non–*Candida albicans* species (e.g., multidrug–resistant *Candida auris*) account for 85% of fungal infections in intensive care units, with a mortality rate of 40–60% (Lombardi et al., 2024; Glavas et al., 2024; Kim et al., 2019); the resistance rate to fluconazole (FLC) continues to rise year by year; *Aspergillus fumigatus* is the primary pathogen responsible for invasive aspergillosis, with a mortality rate of 80% in immunocompromised patients infected with it (Lockhart et al., 2023); in addition, dematiaceous fungi (e.g., *Exophiala dermatitidis*) and *Cryptococcus neoformans* also pose significant challenges to treatment due to their low drug susceptibility (Thambugala et al., 2024; Tio et al., 2023; Kumari et al., 2021).

Currently, there are obvious limitations in the diagnosis and treatment of IFIs: Clinically, there are only 4 classes of antifungal drugs (polyenes, azoles, echinocandins, and 5–fluorocytosine), among which polyenes have strong toxicity, azole resistance is prominent, and echinocandins are ineffective against *Cryptococcu*s (Carmo et al., 2023; Sanyal and Haldar, 2025); at the same time, azole–resistant *A. fumigatus* and multidrug–resistant *C. auris* continue to emerge. Coupled with the high cost of diagnosis and treatment (with annual expenditures of hundreds of millions of US dollars in the United States), the predicament is further exacerbated (Benedict et al., 2019).

Azole drugs [such as FLC, itraconazole (ITR), and voriconazole (VOR)] are core agents for the clinical treatment of fungal diseases. Their target of action is lanosterol 14α–demethylase (CYP51) in the fungal cell membrane—by inhibiting CYP51, they block ergosterol synthesis, and at the same time cause the accumulation of toxic methylated sterols in the cell membrane, disrupting the membrane structure (Chi et al., 2023; Derkacz et al., 2023; Castro-Rios et al., 2025).

Specifically, after azole drugs inhibit ergosterol synthesis, abnormal sterols (such as 14α–demethyl lanosterol) accumulate in the cell membrane, leading to a decrease in membrane fluidity and an increase in permeability, which in turn impairs the integrity of the cell membrane (this also includes mitochondrial membranes) (Feng et al., 2025; Eldesouky et al., 2020; Kolic and Sinko, 2024). The mitochondrial inner membrane serves as the essential scaffold for respiratory chain complexes. Damage to its structural integrity directly impairs the mitochondrial electron transport chain process, leading to electron leakage; the leaked free electrons react with intracellular oxygen, ultimately inducing excessive production of reactive oxygen species (ROS) (Zorov et al., 2014; Liu S. et al., 2025; Rauf et al., 2023). ROS serve as a crucial intermediate during the disruption of mitochondrial integrity induced by azoles, ultimately triggering programmed cell death in fungi (Gonzalez-Jimenez et al., 2023). Therefore, drugs with properties similar to those of azoles, particularly those capable of inducing ROS production and altering cell membrane permeability, may represent promising antifungal agents or adjuvants for azole therapy.

Exploring new antifungal strategies, improving the efficacy of existing drugs, and reversing drug resistance have become core directions in the field. Natural products, characterized by multiple molecular targets and a low propensity to induce drug resistance, have demonstrated remarkable potential in augmenting the antifungal efficacy of azoles, and represent a largely untapped valuable source of azole adjuvants (Kane and Carter, 2022; Ayaz et al., 2019).

Herein, we demonstrate that betulinic acid (BA) possesses both bactericidal and antifungal activities against several fungal and bacterial species, representing a promising candidate for antifungal drug development, although large-scale validation across a comprehensive pathogen panel remains to be performed (Hamion et al., 2024; Rodrigues et al., 2023). Previous studies have shown that BA upregulates intracellular ROS levels via the mitochondrial pathway, and it has also been reported to induce alterations in mitochondrial membrane permeability following treatment (Fulda, 2008; Przystupski et al., 2019). We therefore hypothesize that BA may augment the antifungal efficacy of azoles against pathogenic fungi through the modulation of ROS generation.

## 2 Materials and methods

### 2.1 Fungal strains, antifungals, and chemical agents

In this study, 110 clinically isolated strains were included, consisting of 52 *Aspergillus* strains (27 *A. fumigatus*, 5 *A. niger*, 12 *A. flavus*, and 8 *A. terreus*), 9 *Cryptococcus neoformans* strains, 28 *Candida strain*s (9 *C. albicans*, 10 *C. auris*, 5 *C. glabrata*, 2 *C. tropicalis*, and 2 *C. parapsilosis*), and 21 *E. dermatitidis* strains (Supplementary Table S1) (Sun et al., 2022). All fungal strains were identified by microscopic morphology and by molecular sequencing of the internal transcribed spacer (ITS) ribosomal DNA (rDNA). For identification of *Aspergillus* spp., additional molecular sequence of *β*-tubulin and calmodulin were required (Supplementary Table S2) (Sun et al., 2022). All the sequences of the analyzed strains were uploaded to GenBank (PP069948–PP070390).

These broth microdilution experiments were also performed with *C. parapsilosis* ATCC 22019 and *A. flavus* ATCC 204304 to ensure the accuracy of minimum inhibitory concentration (MIC) determination and exclude experimental errors.

All strains required for this experiment were cultured on Sabouraud Dextrose Agar (SDA, Haibo Biological, Qingdao, China) at 35 °C: *A. niger* was cultured for 3 days, while the remaining strains were cultured for 5 days. Subsequently, susceptibility testing was performed to ensure the viability and purity of the tested fungi.

The experimental drugs, such as ITR (HY–17514, purity = 99.59%), posaconazole (POS, HY–17373, purity = 99.95%) and BA (HY–N1451, purity = 98.77%), were obtained from MedChemExpress (MCE), NJ, USA. VOR (V129745, purity ≥ 98.00%) and FLC (E129360, purity ≥ 98.00%) were purchased from Aladdin Biotech Co., Ltd., China.

All drugs were solubilized in dimethyl sulfoxide (DMSO), with the stock solution concentration standardized at 6,400 μg/mL. For ITR, VOR, and FLC, the working concentration ranges were set at 0.0625–8 μg/mL; POS was applied within the concentration spectrum of 0.03125–4 μg/mL; and BA was utilized at concentrations spanning from 1 to 64 μg/mL. The maximum concentration of DMSO does not exceed 0.1%.

### 2.2 *In vitro* interactions of BA and azoles against pathogenic fungi

Susceptibility testing was performed according to the broth microdilution chequerboard procedure based on the CLSI M27–A3 and M38–A2 standard and previously published protocols (Sun et al., 2022). For yeasts, conidia harvested from cultures grown for 2 days on SDA agar were suspended in sterile distilled water containing 0.03% Triton X-100 (Yeasen, China) and diluted to a concentration of 1–5 × 10^6^ CFU/mL, which were then diluted 1,000 times in RPMI 1640 medium (supplemented with 0.2% (w/v) glucose and 0.165 M 3-(N-morpholino)propanesulfonic acid [MOPS] buffer, pH 7.0; Procell, China) to achieve a 2–fold suspension more concentrated than the density needed or to approximately 2–4 × 10^3^ CFU/mL. For filamentous fungi, conidia harvested from cultures grown for 3 days (*Aspergillus* spp.) or 5 days (*E. dermatitidis*) on SDA were suspended in sterile distilled water containing 0.03% Triton X-100 and diluted to a concentration of 2–5 × 10^6^ CFU/mL, which were then diluted 100 times in RPMI 1640 to achieve a 2–fold suspension more concentrated than the density needed or to approximately 1–3 × 10^4^ CFU/mL. The working concentration ranges of BA, ITR, VOR, POS, and FLC were 1–64 μg/mL, 0.0625–8 μg/mL, 0.0625–8 μg/mL, 0.03125–4 μg/mL, and 0.0625–8 μg/mL, respectively. As described, 50 μL of BA with serial dilutions were inoculated in horizontal direction and another 50 μL of azoles with serial dilutions were inoculated in vertical direction on the 96–well plate, which contained 100 μL prepared inoculum suspension. Interpretation of results was performed after incubation at 35 °C for 24 h for *Candida* spp., 48 h for *C. neoformans*, *Aspergillus* spp., and 72 h for *E. dermatitidis*, respectively. The MICs applied for the evaluation of effects against *Candida* spp. and *C. neoformans* complex were determined as the lowest concentration resulting in 50% inhibition of growth. The MICs applied for the evaluation of effects against *E. dermatitidis* and *Aspergillus* spp. were determined as the lowest concentration resulting in 100% inhibition of growth. The combination interaction between BA and azoles was classified on the basis of the fractional inhibitory concentration index (FICI). The FICI as calculated by the formula: FICI = (Ac/Aa) + (Bc/Ba), where Ac and Bc are the MICs of antifungal drugs in combination, and Aa and Ba are the MICs of antifungal drugs A and B alone. A FICI of ≤ 0.5 is classified as synergy, an FICI of >0.5 to ≤4 indicates no interaction (indifference), and an FICI of >4 indicates antagonism (Sun et al., 2022). All tests were performed in triplicate.

### 2.3 2,7–dichlorofluorescin diacetate (DCFH–DA) staining

Fresh conidia were collected from SDA. With the exception of *E. dermatitidis*, which required 5 days of culture, the other strains were cultured for 3 days. The conidia were divided into 4 treatment groups: no POS or BA added; only POS added (final concentration: half its MIC); only BA added (final concentration: half its MIC); both POS and BA added (each at a final concentration of half their respective MICs when used in combination). The conidia of each group were separately transferred to SAB liquid medium and cultured with shaking at 37 °C and 130 rpm for 36 h. After cultivation, the fungal suspension was collected and washed 5 times with PBS. Subsequently, DCFH–DA (Yeasen, China; concentration 10 μM) was added to each group of samples at a volume ratio of 0.1%, and the mixture was incubated at 37 °C for 30 min. Following incubation, centrifugation was performed at 4000 rpm for 20 min, the supernatant was discarded, and the pellet was retained. Data were acquired using Beckman Cytomics FC 500 and BD FACSCanto II flow cytometers and analyzed with FlowJo v10 software. The excitation wavelength of the experiment was 488 nm, and the emission wavelength was 525 nm.

### 2.4 Data processing software

GraphPad Prism 9 software was used for mapping and used for statistical analysis and mean ± standard deviation (SD) was used for data representation. Single factor analysis of variance (ANOVA) was used, and the difference was statistically significant (*p* < 0.05). Correlation analysis was performed using Spearman’s correlation test. The 95% confidence intervals (95% CIs) of synergy and antagonism rates were calculated using the Wilson score interval method.

## 3 Results

### 3.1 *In vitro* interactions between BA and azoles against *Aspergillus* spp.

When BA was used alone, the MIC of all *Aspergillus* strains exceeded 64 μg/mL, except for *A. fumigatus* AF20 and *A. flavus* 3,357. For azole drugs used alone, their MIC ranges against *Aspergillus* spp. were as follows: ITR, 0.125–4 μg/mL; VOR, 0.125–4 μg/mL; and POS, 0.0625–1 μg/mL (Table 1).

**Table 1:** *In vitro* drug sensitization results of BA combined with azoles against *Aspergillus* spp.

Strains	MIC alone (μg/mL)				MIC combinations (μg/mL)		
	BA	ITR	VOR	POS	BA/ITR	BA/VOR	BA/POS
*A. fumigatus*							
AF1	>64	1	0.25	0.25	2/0.25(S)	2/0.25(I)	2/0.063(S)
AF2	>64	0.5	0.25	0.25	8/0.5(I)	2/0.25(I)	2/0.031(S)
AF3	>64	0.5	0.25	0.25	16/0.5(I)	2/0.25(I)	1/0.031(S)
AF4	>64	1	0.25	0.25	8/0.5(I)	2/0.25(I)	2/0.031(S)
AF5	>64	0.5	0.25	0.25	32/8(A)	2/0.25(I)	2/0.063(S)
AF6	>64	0.5	0.25	0.25	32/8(A)	2/0.25(I)	2/0.063(S)
AF7	>64	4	2	1	4/4(I)	2/2(I)	1/0.5(I)
AF8	>64	0.5	0.25	0.25	32/8(A)	2/0.25(I)	4/0.031(S)
AF9	>64	0.25	0.125	0.25	8/0.5(I)	2/0.125(I)	32/0.031(S)
AF10	>64	0.5	0.5	0.25	8/0.5(I)	16/0.5(I)	1/0.063(S)
AF11	>64	0.5	0.25	0.25	8/0.5(I)	2/0.25(I)	2/0.031(S)
AF12	>64	0.5	0.125	0.25	16/2(A)	8/0.25(I)	4/0.031(S)
AF13	>64	0.5	0.25	0.25	8/0.5(I)	2/0.25(I)	1/0.063(S)
AF14	>64	0.5	0.25	0.25	8/0.5(I)	2/0.25(I)	2/0.063(S)
AF15	>64	0.5	4	1	8/1(I)	2/4(I)	1/1(I)
AF16	>64	0.5	0.25	0.125	16/4(A)	2/0.25(I)	1/0.031(S)
AF17	>64	0.5	0.5	0.25	16/4(A)	2/0.5(I)	2/0.063(S)
AF18	>64	0.5	0.125	0.125	8/0.5(I)	2/0.125(I)	1/0.031(S)
AF19	>64	0.5	0.25	0.25	8/0.5(I)	2/0.25(I)	2/0.063(S)
AF20	16	0.25	0.125	0.125	32/1(A)	2/0.125(I)	16/0.031(I)
AF21	>64	0.5	0.25	0.125	8/0.5(I)	2/0.25(I)	2/0.031(S)
AF22	>64	0.25	0.125	0.063	32/1(A)	2/0.125(I)	1/0.031(I)
AF23	>64	0.5	0.125	0.063	8/0.5(I)	2/0.125(I)	1/0.031(I)
AF24	>64	0.5	0.25	0.25	16/2(A)	2/0.25(I)	1/0.031(S)
AF25	>64	0.5	0.125	0.125	8/0.5(I)	2/0.125(I)	1/0.031(S)
AF26	>64	0.5	0.25	0.25	16/2(A)	2/0.25(I)	1/0.031(S)
AF27	>64	0.5	0.25	0.125	32/8(A)	2/0.25(I)	1/0.031(S)
*A. flavus*							
AL1	>64	0.5	0.5	0.25	16/1(I)	32/1(I)	1/0.125(I)
AL2	>64	0.25	0.25	0.25	8/0.5(I)	4/0.5(I)	1/0.031(S)
AL3	>64	0.125	0.25	0.5	16/1(A)	2/0.25(I)	1/0.125(S)
AL4	>64	0.5	2	0.5	8/1(I)	2/2(I)	1/0.25(I)
AL5	>64	0.5	0.5	0.25	1/0.25(I)	2/0.5(I)	1/0.063(S)
AL6	>64	0.5	1	0.25	16/2(A)	16/2(I)	2/0.031(S)
AL7	>64	0.5	0.25	0.5	8/1(I)	2/0.25(I)	1/0.125(S)
AL8	>64	0.5	0.5	0.5	1/0.25(I)	2/0.5(I)	1/0.25(I)
AL9	>64	0.5	2	0.25	8/1(I)	2/2(I)	1/0.063(S)
AL10	>64	0.5	0.5	0.25	1/0.25(I)	2/0.5(I)	1/0.063(S)
NRRL 3357	64	0.5	0.5	0.5	2/0.5(I)	16/1(I)	2/0.125(S)
ATCC 204304	>64	0.5	1	0.5	8/0.5(I)	2/1(I)	2/0.125(S)
*A. terreus*							
AT1	>64	0.25	0.25	0.25	1/0.125(I)	2/0.25(I)	1/0.063(S)
AT2	>64	0.25	0.125	0.25	1/0.063(S)	2/0.25(I)	1/0.063(S)
AT3	>64	0.25	0.25	0.25	1/0.125(I)	2/0.25(I)	1/0.063(S)
AT4	>64	0.25	0.125	0.125	2/0.063(S)	2/0.125(I)	1/0.031(S)
AT5	>64	0.125	0.25	0.125	16/0.5(A)	2/0.25(I)	1/0.031(S)
AT6	>64	0.5	0.5	0.125	4/0.125(S)	2/0.5(I)	1/0.031(S)
AT7	>64	0.5	0.25	0.25	1/0.125(S)	2/0.25(I)	2/0.031(S)
AT8	>64	0.5	0.5	0.25	4/0.125(S)	2/0.5(I)	1/0.063(S)
*A. niger*							
AN1	>64	0.5	0.5	0.25	8/1(I)	2/0.5(I)	1/0.125(I)
AN2	>64	0.5	0.5	0.25	8/1(I)	2/0.5(I)	1/0.063(S)
AN3	>64	0.5	0.5	0.5	4/1(I)	2/0.5(I)	4/0.125(S)
AN4	>64	1	0.5	0.5	1/0.5(I)	2/0.5(I)	2/0.125(S)
AN5	>64	0.5	0.5	0.25	8/1(I)	2/0.5(I)	1/0.063(S)

ITR, itraconazole; VOR, voriconazole; POS, posaconazole; BA, betulinic acid; S, synergy (FICI ≤0.5); I, indifference (no interaction, FICI from >0.5 to ≤4); A, antagonism (FICI of >4). MICs were the concentrations that achieved 100% growth inhibition; FICI: fractional inhibitory concentration index.

When BA was combined with ITR, VOR, and POS respectively, synergistic effects were observed in 6 (11.5%), 0 (0.00%), and 43 (82.7%) strains. Antagonistic effects were only found in the BA–ITR combination, in 14 (26.9%) strains (Supplementary Figure S1).

In the synergistic combinations, the MICs of BA and POS against *Aspergillus* spp. decreased to 1–32 μg/mL and 0.03125–1 μg/mL, respectively (Table 1), with 48 strains showing a BA MIC of 1–2 μg/mL. Specifically, synergistic effects were detected in 22/27 (81.5%) *A. fumigatus* strains, 9/12 (75%) *A. flavus* strains, 4/5 (80%) *A. niger* strains, and 8/8 (100%) *A. terreus* strains (Table 1).

### 3.2 *In vitro* interactions between BA and azoles against *Exophiala dermatitidis*

When used alone, the MIC ranges of BA, ITR, VOR and POS *E. dermatitidis* were as follows: >32 μg/mL, 0.0625–0.5 μg/mL, 0.0625–0.5 μg/mL and 0.125–2 μg/mL, respectively (Table 2).

**Table 2:** *In vitro* drug sensitization results of BA combined with azoles against *E. dermatitidis.*

Strains	MIC alone (μg/mL)				MIC combinations (μg/mL)		
	BA	ITR	VOR	POS	BA/ITR	BA/VOR	BA/POS
BMU00028	>32	0.063	0.063	0.25	2/0.063(I)	2/0.063(I)	1/0.063(S)
BMU00029	>32	0.5	0.25	0.25	2/0.5(I)	2/0.25(I)	1/0.063(S)
BMU00030	>32	0.25	0.25	0.5	2/0.25(I)	2/0.25(I)	2/0.125(S)
BMU00031	>32	0.25	0.125	0.25	16/0.5(I)	2/0.125(I)	1/0.063(S)
BMU00034	>32	0.25	0.125	0.5	16/0.5(I)	2/0.125(I)	2/0.125(S)
BMU00035	>32	0.25	0.125	0.5	32/2(A)	2/0.125(I)	1/0.125(S)
BMU00036	>32	0.25	0.125	0.5	2/0.25(I)	2/0.125(I)	1/0.125(S)
BMU00037	>32	0.063	0.063	0.25	2/0.063(I)	2/0.063(I)	1/0.063(S)
BMU00038	>32	0.25	0.125	0.5	16/0.5(I)	2/0.125(I)	4/0.125(S)
BMU00039	>32	0.5	0.5	0.25	2/0.5(I)	2/0.5(I)	2/0.063(S)
BMU00040	>32	0.125	0.063	0.25	8/0.25(I)	2/0.125(I)	1/0.063(S)
BMU00041	>32	0.25	0.25	0.5	2/0.25(I)	16/0.5(I)	1/0.125(S)
NPRC 3.8.653	>32	0.063	0.25	0.5	2/0.063(I)	2/0.25(I)	1/0.125(S)
NPRC 3.8.654	>32	0.25	0.063	0.5	2/0.25(I)	2/0.063(I)	1/0.125(S)
NPRC 3.8.655	>32	0.063	0.063	0.25	2/0.063(I)	2/0.063(I)	4/0.063(S)
NPRC 3.8.656	>32	0.25	0.25	0.125	2/0.25(I)	2/0.25(I)	4/0.031(S)
109140	>32	0.5	0.25	0.125	2/0.5(I)	2/0.25(I)	2/0.031(S)
109144	>32	0.25	0.125	0.25	2/0.25(I)	2/0.125(I)	2/0.063(S)
109145	>32	0.25	0.125	0.5	2/0.25(I)	2/0.125(I)	1/0.125(S)
109148	>32	0.063	0.25	0.125	2/0.063(I)	2/0.25(I)	1/0.031(S)
109152	>32	0.25	0.063	2	2/0.25(I)	2/0.063(I)	16/4(I)

ITR, itraconazole; VOR, voriconazole; POS, posaconazole; BA, betulinic acid; S, synergy (FICI ≤0.5); I, indifference (no interaction, FICI from >0.5 to ≤4); A, antagonism (FICI of >4). MICs were the concentrations that achieved 100% growth inhibition; FICI: fractional inhibitory concentration index.

When BA was combined with ITR, VOR or POS, synergistic effects were only observed in the BA–POS combination, with a high synergy rate of 95.2% (20/21). In this synergistic combination, the MICs of BA and POS decreased to 1–4 μg/mL and 0.03125–0.125 μg/mL, respectively. Notably, a synergistic effect was detected in 1 (4.8%) strain in the BA–ITR combination (Supplementary Figure S2).

### 3.3 *In vitro* interactions between BA and azoles against *Candida* spp.

FLC is the first-line therapeutic agent for most *Candida* infections. Given that *C. auris* displays widespread intrinsic resistance to FLC, which renders susceptibility testing of this agent clinically irrelevant for this species, FLC was evaluated against all *Candida* species except *C. auris* (Akwongo et al., 2024; Song et al., 2026). When used alone, the MIC ranges of BA, ITR, VOR and POS were >64 μg/mL, 0.625–4 μg/mL, 0.625–4 μg/mL and 0.03125–1 μg/mL, respectively. For non–*Candida auris* strains, the MIC range of FLC was 0.25–8 μg/mL (Table 3).

**Table 3:** *In vitro* drug sensitivity results of BA combined with azoles against *Candida* spp.

Strains	MIC alone (μg/mL)					MIC combinations (μg/mL)			
	BA	ITR	VOR	FLC	POS	BA/ITR	BA/VOR	BA/FLC	BA/POS
*C. albicans*									
R8	>64	0.063	0.063	8	0.031	32/0.25(A)	2/0.063(I)	2/8(I)	64/0.063(I)
R9	>64	0.125	0.063	2	0.063	16/1(A)	4/0.25(A)	1/8(A)	4/0.25(A)
R14	>64	0.125	0.125	2	0.031	8/0.125(I)	2/0.125(I)	32/2(I)	2/0.031(I)
R66	>64	0.25	0.25	8	0.063	1/0.063(S)	2/0.25(I)	2/8(I)	32/0.063(I)
122473	>64	0.063	0.063	1	0.125	64/0.5(A)	2/0.063(I)	2/1(I)	2/0.125(I)
129968	>64	0.125	0.063	0.25	0.031	32/0.5(A)	2/0.063(I)	8/0.125(I)	2/0.031(I)
122843	>64	0.063	0.063	0.25	0.031	32/0.125(I)	2/0.063(I)	8/0.5(I)	2/0.031(I)
123118	>64	0.063	0.063	0.25	0.031	2/0.063(I)	2/0.063(I)	2/0.25(I)	2/0.031(I)
130110	>64	0.063	0.063	0.25	0.031	2/0.063(I)	2/0.063(I)	2/0.25(I)	2/0.031(I)
*C. auris*									
381	>64	0.25	0.125	ND	0.125	32/1(A)	2/0.125(I)	ND	16/0.25(I)
382	>64	0.063	0.063		1	2/0.063(I)	2/0.063(I)		2/0.25(S)
383	>64	0.063	0.063		0.25	64/0.25(A)	2/0.063(I)		2/0.031(S)
384	>64	0.25	2		0.25	2/0.063(S)	2/2(I)		4/0.031(S)
385	>64	0.25	1		0.5	2/0.063(S)	2/1(I)		2/0.125(S)
386	>64	0.5	4		0.125	1/0.25(I)	8/1(S)		1/0.031(S)
387	>64	0.063	0.063		0.031	2/0.063(I)	2/0.063(I)		1/0.031(I)
388	>64	0.5	2		0.125	32/0.5(I)	16/0.5(S)		8/0.031(S)
389	>64	0.25	1		0.125	2/0.25(I)	2/1(I)		2/0.125(I)
390	>64	0.5	1		0.125	2/0.125(S)	8/0.25(S)		1/0.031(S)
*C. glabrata*									
122512	>64	2	0.25	4	0.5	32/8(A)	2/0.25(I)	1/8(I)	64/4(A)
122889	>64	2	0.125	4	0.5	16/8(A)	1/0.25(I)	2/4(I)	64/2(A)
122127	>64	1	0.063	2	0.25	16/8(A)	16/0.25(A)	2/8(A)	16/1(A)
130133	>64	0.125	0.063	4	0.125	16/0.5(A)	8/0.25(A)	32/4(I)	2/0.125(I)
5448	>64	0.25	0.063	2	0.125	2/0.25(I)	8/0.25(A)	1/4(I)	16/0.25(I)
*C. tropicalis*									
122115	>64	0.25	0.125	4	0.125	1/0.063(S)	2/0.125(I)	2/4(I)	2/0.031(S)
122543	>64	0.125	0.063	0.25	0.031	32/0.5(A)	2/0.063(I)	2/0.25(I)	64/0.063(I)
*C. parapsilosis*									
117406	>64	0.063	0.063	2	0.031	64/0.125(I)	2/0.063(I)	2/8(A)	2/0.031(I)
22019	>64	0.125	0.063	1	0.031	1/0.125(I)	2/0.063(I)	1/0.5(I)	2/0.031(I)

ITR, itraconazole; VOR, voriconazole; POS, posaconazole; FLC, fluconazole; BA, betulinic acid; S, synergy (FICI ≤ 0.5); I, indifference (no interaction, FICI from >0.5 to ≤4); A, antagonism (FICI of >4); ND, Not Determined. MICs were the concentrations that achieved 50% growth inhibition; FICI: fractional inhibitory concentration index.

Among non–*C. auris* strains, when BA was combined with ITR, VOR, POS, and FLC, synergistic effects were observed in 2 (11.1%) strains, 0 strains, 1 (5.6%) strain and 0 strains, respectively; antagonistic effects were observed in 9 strains (50%), 4 (22.2%) strains, 4 (22.2%) strains and 3 (16.7%) strains, respectively. For *C. auris* strains, the combination of BA with ITR, VOR, and POS resulted in synergistic effects in 3 (30%) strains, 3 (30%) strains and 7 (70%) strains, respectively; 2 (20%) strain exhibited antagonism with ITR (Supplementary Figure S3).

In the synergistic combination of BA and ITR, their MICs were reduced to 1–2 μg/mL and 0.0625–0.125 μg/mL, respectively (Table 3). When BA was combined with VOR, the effective MIC ranges of the two agents were decreased to 8–16 μg/mL and 0.25–1 μg/mL, respectively. For the combination of BA and POS, the effective concentration ranges of the two agents were 1–4 μg/mL and 0.03125–0.25 μg/mL, respectively (Table 3).

Notably, among the 10 *C. auris* strains tested, 7 (70%) strains showed a combined effect with POS, and the MIC of azole agents was reduced by 4–8 folds.

### 3.4 *In vitro* interactions between BA and azoles against *Cryptococcus neoformans*

When used alone, the MIC ranges of BA, ITR, VOR, POS and FLC against *C. neoformans* were as follows: >64 μg/mL, 0.125–0.25 μg/mL, 0.0625 μg/mL, 0.125–0.25 μg/mL, and 0.25–2 μg/mL, respectively (Table 4).

**Table 4:** *In vitro* drug sensitivity results of BA combined with azoles against *C. neoformans.*

Strains	MIC alone (μg/mL)					MIC combinations (μg/mL)			
	BA	ITR	VOR	FLC	POS	BA/ITR	BA/VOR	BA/FLC	BA/POS
Y1	>64	0.125	0.063	2	0.125	8/0.5(A)	4/0.25(A)	1/8(A)	1/0.25(I)
Y2	>64	0.25	0.063	2	0.125	8/1(A)	2/0.25(A)	2/8(A)	16/0.5(A)
Y3	>64	0.125	0.063	1	0.125	4/0.125(I)	1/0.125(I)	8/0.25(S)	1/0.063(I)
Y6	>64	0.125	0.063	2	0.125	2/0.063(I)	2/0.063(I)	16/0.5(S)	2/0.063(I)
Y7	>64	0.125	0.063	1	0.125	4/0.125(I)	1/0.125(I)	8/1(I)	1/0.063(I)
Y8	>64	0.125	0.063	1	0.125	16/0.031(S)	16/0.063(I)	8/0.125(S)	16/0.031(S)
Y9	>64	0.25	0.063	2	0.125	4/0.125(I)	1/0.125(I)	16/1(I)	4/0.063(I)
Y11	>64	0.125	0.063	0.25	0.125	32/0.063(I)	4/0.063(I)	32/0.063(S)	1/0.063(I)
Y12	>64	0.125	0.063	1	0.25	4/0.125(I)	4/0.25(A)	8/0.25(S)	1/0.063(S)

ITR, itraconazole; VOR, voriconazole; POS, posaconazole; FLC, fluconazole; BA, betulinic acid; S, synergy (FICI ≤ 0.5); I, indifference (no interaction, FICI from >0.5 to ≤4); A, antagonism (FICI of >4). MICs were the concentrations that achieved 50% growth inhibition; FICI: fractional inhibitory concentration index.

When BA was combined with ITR, VOR, POS, or FLC, among the isolated *C. neoformans* strains: synergistic effects were observed in 1 (11.1%), 0 (0%), 2 (22.2%), and 5(55.6%) strains, respectively; while antagonistic effects were detected in 2 (22.2%), 3 (33.3%), 1 (11.1%), and 2 (22.2%) strains, respectively (Supplementary Figure S4).

In the BA–FLC combination (which showed the best synergistic effect), the MICs of BA and FLC decreased to 8–32 μg/mL and 0.0625–0.5 μg/mL, respectively (Table 4).

### 3.5 Summary of *in vitro* interaction between BA and azole drugs against pathogenic fungi

Among the 52 *Aspergillus* strains: the BA/ITR combination showed synergistic effects on 6 (11.5%) strains, and the BA/POS combination on 43 (82.7%) strains; meanwhile, the BA/ITR combination induced antagonistic effects on 14 (26.9%) strains (Table 5).

**Table 5:** Summary of drug interaction for the combination of BA and azoles.

Species(*n*)	*n* (%) of isolates showing synergism or antagonism for the combination							
	BA+ITR		BA+VOR		BA+POS		BA+FLC	
	S	A	S	A	S	A	S	A
*Aspergillus* spp. (52)	6(11.5%)	14(26.9%)	0	0	43 (82.7%)	0	ND	
*A. fumigatus* (27)	1 (3.7%)	11 (40.7%)	0	0	22 (81.5%)	0		
*A. flavus* (12)	0	2 (16.7%)	0	0	9 (75.0%)	0		
*A. terreus* (8)	5 (62.5%)	1 (12.5%)	0	0	8 (100.0%)	0		
*A. niger* (5)	0	0	0	0	4 (80.0%)	0		
*Candida* spp. (28)	5 (17.9%)	11 (39.3%)	3 (10.7%)	4 (14.3%)	8 (28.6%)	4 (14.3%)	0	3 (10.7%)
*C. albicans* (9)	1 (11.1%)	4 (44.4%)	0	1 (11.1%)	0	1 (11.1%)	0	1 (11.1%)
*C. auris* (10)	3 (30.0%)	2 (20.0%)	3 (30.0%)	0	7 (70.0%)	0	ND	
*C. glabrata* (5)	0	4 (80.0%)	0	3 (60.0%)	0	3 (60.0%)	0	1 (20.0%)
*C. tropicalis* (2)	1 (50.0%)	1 (50.0%)	0	0	1 (50.0%)	0	0	0
*C. parapsilosis* (2)	0	0	0	0	0	0	0	1 (50.0%)
*E. dermatitidis* (21)	0	1 (4.8%)	0	0	20 (95.2%)	0	ND	
*C. neoformans* (9)	1 (11.1%)	2 (22.2%)	0	3 (33.3%)	2 (22.2%)	1 (11.1%)	5 (55.6%)	2 (22.2%)
Total (110)	12 (10.9%)	28 (25.5%)	3 (2.7%)	7 (6.4%)	73 (66.4%)	5 (4.5%)	5 (18.5%)	5 (18.5%)
95% CI	6.4%–18.1%	18.2%–34.3%	0.7%–7.7%	3.1%–12.6%	57.1%–74.5%	2.0%–10.2%	8.2%–36.7%	8.2%–36.7%

ITR, itraconazole; VOR, voriconazole; POS, posaconazole; BA, betulinic acid; S, synergy (FICI ≤ 0.5); I, indifference (no interaction, FICI from >0.5 to ≤4); A, antagonism (FICI of >4), ND, Not Determined. MICs were the concentrations that achieved 100% growth inhibition; FICI, fractional inhibitory concentration index; 95% CI, 95% confidence interval calculated by the Wilson score interval method.

Among the 21 *E. dermatitidis* strains: the BA/POS combination exhibited synergistic effects on 20 (95.2%) strains, and the BA/ITR combination showed an antagonistic effect on 1 (4.8%) strain (Table 5).

Among the 9 *C. neoformans* strains: the BA/ITR, BA/POS and BA/FLC combinations displayed synergistic effects on 1 (11.1%), 2 (22.2%) and 5 (55.6%) strains, respectively; antagonistic effects were observed in the BA/ITR combination (2 strains, 22.2%), BA/VOR combination (3 strains, 33.3%), BA/POS combination (1 strain, 11.1%), and BA/FLC combination (2 strains, 22.2%) (Table 5).

Among the 28 *Candida* strains: the BA/ITR, BA/VOR and BA/POS combinations showed combined effects on 5 (17.9%), 3 (10.7%), and 8 (28.6%) strains, respectively; antagonistic effects were detected in 11 (39.2%), 4 (14.3%) and 4 (14.3%) strains. The BA/FLC combination showed no synergistic effect on non–*C. auris* strains; notably, strains exhibiting synergism with POS were mainly *C. auris*—7 out of 10 (70%) *C. auris* strains showed synergistic effects (Table 5).

**Figure 1:**
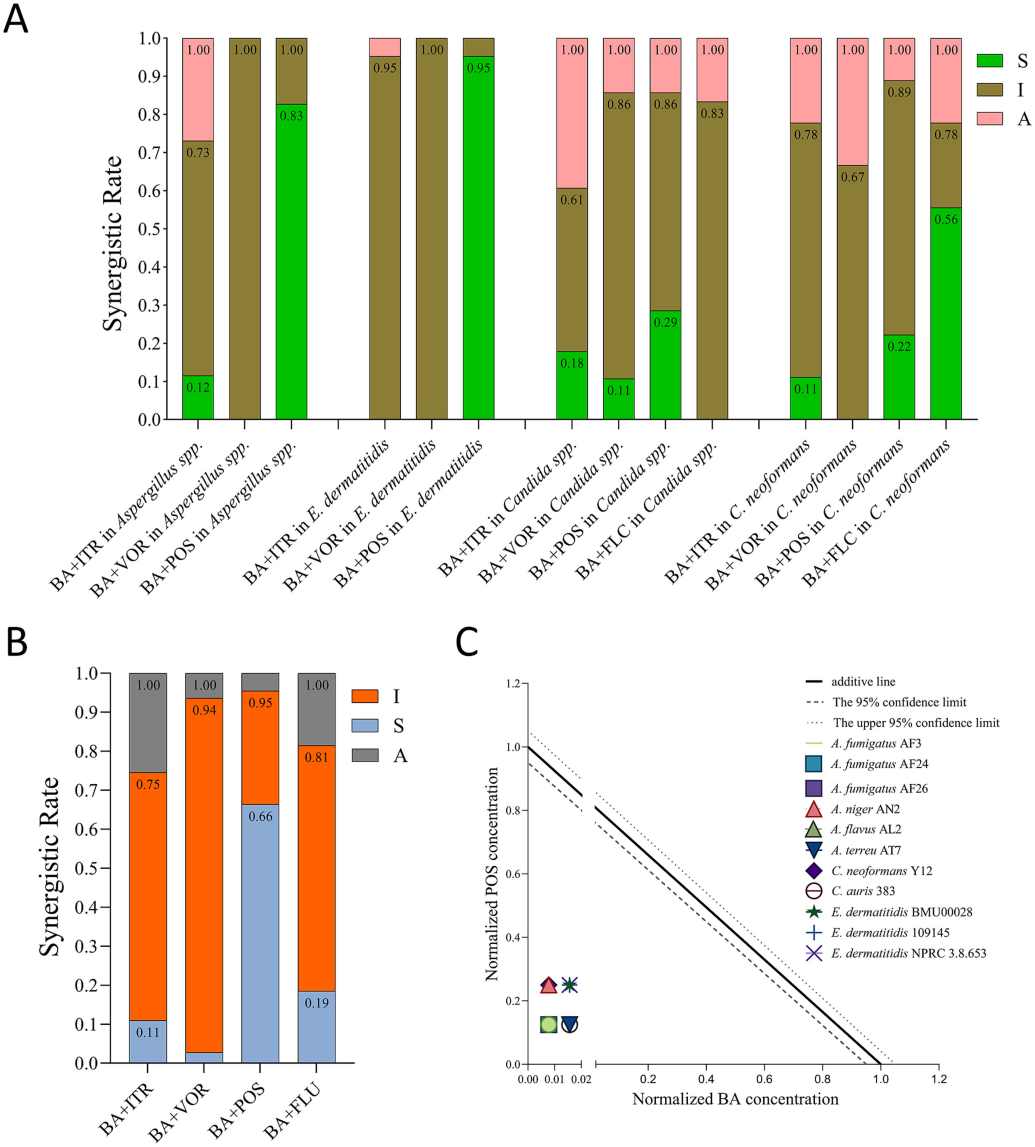
Summary of drug interaction for the combination of BA and azoles. **(A)** Species–specific summary of interactions between BA–azole combinations and pathogenic fungi. **(B)** Azole–specific summary of interactions between BA–azole combinations and pathogenic fungi. **(C)** Isobologram analysis of the synergistic interaction between BA and POS. The X-axis represents normalized POS concentration, and the Y-axis represents normalized BA concentration. Normalized concentration was calculated as the MIC of the drug in combination divided by the MIC of the drug alone. The solid diagonal line represents the theoretical additive effect based on the Loewe additivity model. The upper and lower dashed lines indicate the 95% confidence intervals of the additive line. Each symbol corresponds to a different clinical isolate. Data points located below the lower 95% confidence line indicate synergistic activity. S, synergy (FICI≤0.5); I, indifference (FICI from >0.5 to ≤4); A, antagonism (FICI of >4); BA, betulinic acid; ITR, itraconazole; VOR, voriconazole; POS, posaconazole; FLC, fluconazole. The synergism rate was calculated by dividing the number of strains exhibiting synergism by the total number of strains tested.

Classified by azole type, when BA was combined with POS, ITR, VOR and FLC, the synergy rates against all pathogenic fungi were 73/110 (66.4%), 12/110 (10.9%), 3/110 (2.7%) and 5/27 (18.5%), respectively; the antagonism rates were 5/110 (4.5%), 28/110 (25.5%), 7/110 (6.4%), and 5/27 (18.5%), respectively (Figure 1). Isobolographic analysis was performed using representative strains. The results showed that the normalized MIC values of the BA-POS combination for all tested isolates were significantly below the lower 95% confidence limit of the theoretical additive line, indicating strong synergistic interactions. These findings demonstrate that the combination of BA and POS exhibits clear synergistic antifungal activity against a broad spectrum of pathogenic fungi.

### 3.6 Combination of BA and POS significantly increases ROS levels in fungi

To explore the correlation between the synergistic antifungal effect of BA combined with POS and the production level of ROS, a total of 21 strains were selected for ROS detection. Among them, 17 strains were used in the BA plus POS (BA+POS) group. Eleven strains exhibited synergistic effects with low FICI values, including 3 *A. fumigatus* strains (AF3, FICI = 0.133; AF24, FICI = 0.133; AF26, FICI = 0.133), 1 *A. flavus* strain (AL2, FICI = 0.133), 1 *A. niger* strain (AN2, FICI = 0.258), 1 *A. terreus* strain (AT7, FICI = 0.141), 1 *C. auris* strain (383, FICI = 0.141), 1 *C. neoformans* strain (Y12, FICI = 0.258), and 3 *E. dermatitidis* strains (NPRC 3.8.653, FICI = 0.266; BMU00028, FICI = 0.266; 109145, FICI = 0.266). The other 6 strains served as non-synergistic control strains: *A. fumigatus* AF20 (FICI = 1.250), *A. niger* AN1 (FICI = 0.508), *A. flavus* AL1 (FICI = 0.508), *C. neoformans* Y2 (FICI = 4.125), *C. albicans* 381 (FICI = 2.125), and *E. dermatitidis* 109152 (FICI = 2.250). The remaining 4 strains were assigned to the BA plus ITR (BA+ITR) group, namely *A. fumigatus* AF5 (FICI = 16.250), *C. neoformans* Y1 (FICI = 4.063), *C. albicans* R9 (FICI = 8.125), and *E. dermatitidis* BMU00035 (FICI = 8.500).

**Figure 2:**
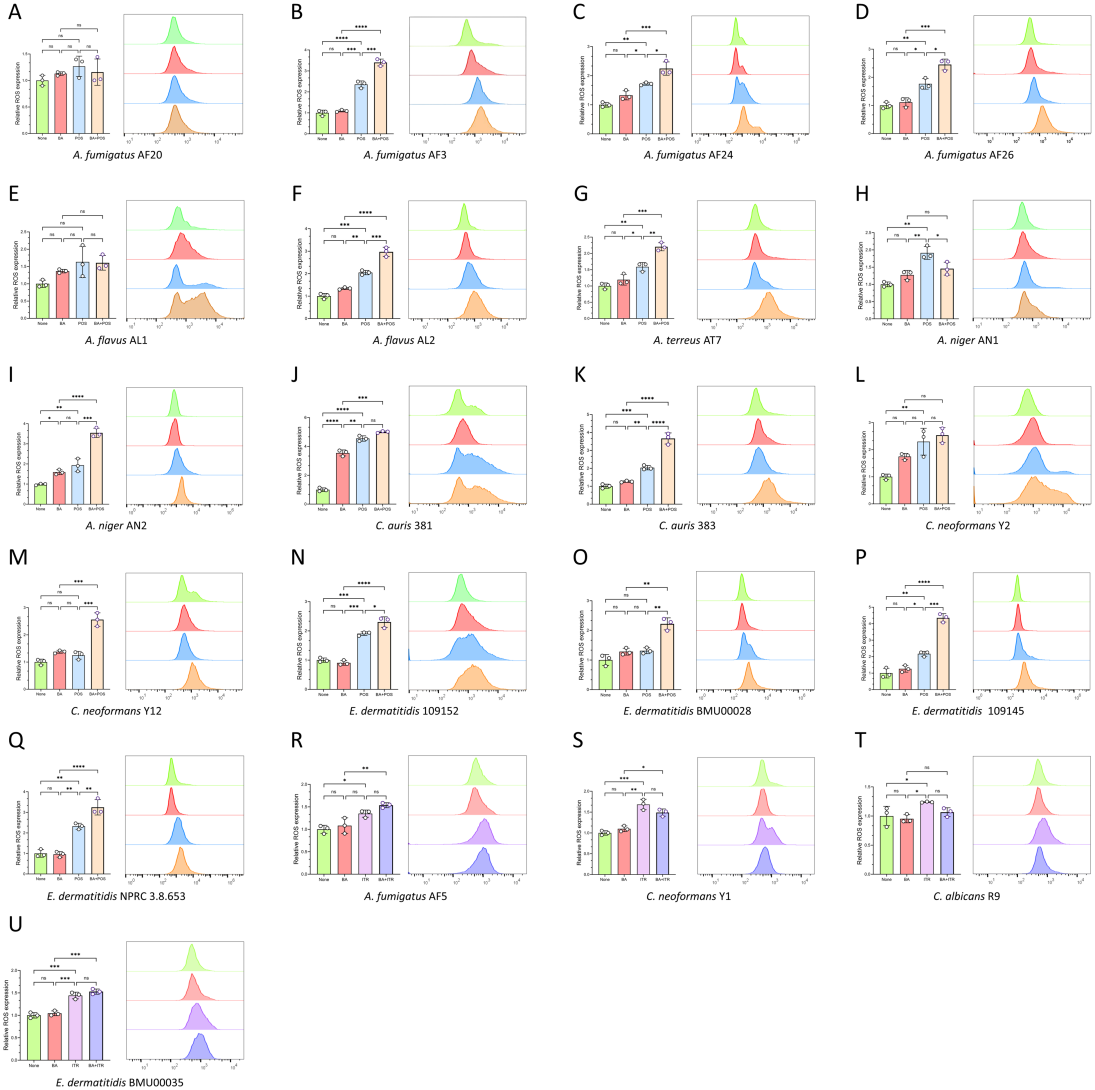
**(A–Q)** Detection of ROS levels after combined treatment with BA and POS; **(R–U)** Detection of ROS levels after combined treatment with BA and ITR. One–way ANOVA followed by Tukey’s HSD test was used for multiple group comparisons. POS, posaconazole; BA, betulinic acid; ns, *p* > 0.05; *p < 0.05; **p < 0.01; ***p < 0.001; ****p < 0.0001.

Among the 11 synergistic strains, the ROS content in the BA/POS combination group was significantly the highest among all treatment groups: compared with the drug-free control group, the ROS level was increased by 2.9–4.8 folds; compared with the BA monotherapy group, it was increased by 2.2–4.4 folds; compared with the POS monotherapy group, it was increased by 1.3–2.0 folds. In contrast, among the 6 non-synergistic strains, compared with the POS monotherapy group, the ROS content in the BA/POS combination group was significantly increased only in *E. dermatitidis* 109152, significantly decreased in *A. niger* AN1, and showed no statistical significance in the other 4 strains. For the 4 strains exhibiting antagonistic effects, the ROS content of the combination group was also comparable to that of the ITR monotherapy group. Notably, the MIC of POS alone against *E. dermatitidis* 109152 was 2 μg/mL, which was much higher than the MIC values of the other 20 strains (0.125–0.5 μg/mL). In addition, ROS levels were significantly elevated in 2 strains after single BA treatment. Treatment with POS alone and ITR alone led to obvious ROS increases in 13 and 4 strains, respectively (Figure 2). Correlation analysis revealed that the ROS levels induced by POS alone and the combination of BA and POS showed a significantly strong positive correlation (*r* = 0.70, *p* < 0.05), whereas no statistically significant correlation was observed between ROS levels of the combination group and FICI values (*r* = 0.25, *p* > 0.05) (Supplementary Figure S5).

## 4 Discussion

This study systematically evaluated the *in vitro* combined antifungal effect of BA combined with four clinically commonly used azole drugs (POS, ITR, VOR, FLC) against 110 clinically isolated pathogenic fungi, including *Aspergillus* spp., *E. dermatitidis*, *Candida* spp., and *C. neoformans*. The results showed that the BA–POS combination exhibited significant synergistic effects against *Aspergillus* spp. and *E. dermatitidis*. The synergy rate reached 82.7% among 52 *Aspergillus* strains, an astonishingly high 95.2% among 21 *E. dermatitidis* strains, and 70% among 10 *C. auris* strains. Additionally, in the BA–POS combination group, the MIC of BA was generally reduced from >64 μg/mL to 0.03125–1 μg/mL, indicating that BA can exert a synergistic effect at low concentrations without potential toxicity risks associated with its independent antifungal activity. By contrast, the synergy rates of BA combined with ITR and VOR were significantly lower (10.9% and 2.7%, respectively), and the BA–ITR combination showed antagonistic effects against 26.9% of *Aspergillus* strains. Compared with POS monotherapy, the intracellular ROS level in the fungal co-culture system was significantly increased after drug combination, indicating that ROS may play a role in the synergistic effect.

BA is mainly used for cancer treatment and prevention (Kamran et al., 2022). It promotes the release of cytochrome c from mitochondria to the cytosol by inducing matrix metalloproteinases, which leads to the generation of ROS, activation of p38 and mitogen–activated protein kinases (MAPKs), and ultimately results in caspase cleavage, initiation of the mitochondrial apoptotic pathway, and nuclear degradation, thereby exerting anticancer activity (Kamran et al., 2022; Chen et al., 2024). Notably, MAPKs are highly conserved in eukaryotes (Jones and Borkovich, 2010); for instance, tyrosine phosphatases related to the MAPK pathway regulate the development, secondary metabolism, and pathogenicity of the fungus *A. flavus* (Yang et al., 2020). This suggests that BA may also affect the physiological processes of fungi.

Antifungal drugs targeting cell membranes, such as ITR, terbinafine and amphotericin B, can elevate intracellular ROS production and thereby exert antifungal effects (Shekhova et al., 2017). Specifically, azole drugs (e.g., POS)—a class of clinically commonly used antifungal agents—target lanosterol 14α–demethylase (Erg11, its homologous protein in mammals is called CYP51) in fungi. This enzyme is the rate–limiting enzyme in the biosynthetic pathway of ergosterol, a key sterol component unique to fungal cell membrane (Alcazar-Fuoli and Mellado, 2013). Azole drugs competitively bind to the heme group at the active site of Erg11, significantly inhibiting its catalytic activity. This inhibition leads to two main consequences: first, the biosynthesis of ergosterol in the cell membrane is blocked, resulting in a continuous depletion of ergosterol content; second, toxic intermediates such as 14α–methyl lanosterol accumulate in large quantities in the membrane. The abnormal composition of membrane sterols directly disrupts the order of the phospholipid bilayer of the cell membrane, causing damage to membrane integrity and a significant increase in permeability (Elsaman et al., 2024; Becher and Wirsel, 2012; Zhang et al., 2019). These changes ultimately lead to cellular metabolic disorders and altered ROS production.

This imbalanced ROS in turn damages the fungal cell membrane, further reducing membrane fluidity and increasing permeability in an additive manner, which exacerbates intracellular metabolic disorders. Oxidative stress mediated by ROS may damage biological macromolecules such as proteins, lipids and nucleic acids, which may be a cause of fungal cell death (Wu et al., 2024; Blagov et al., 2024).

Consistent with previous studies, among the four azole drugs involved in this study, the synergistic antifungal effect of POS combined with BA was the most optimal (Chen et al., 2025; Pinder et al., 2025; Zhang et al., 2025), whereas an antagonism rate of 24.5% was detected in the combination with ITR, which cannot be ignored. As a derivative of ITR, POS has been widely used in the prevention and treatment of invasive aspergillosis, with significantly superior antifungal activity to ITR (Frampton and Scott, 2008). *In vitro* experiments have confirmed that the inhibitory activity of POS against *Candida* spp. is 2–4 times that of ITR, the inhibition rate against *C. neoformans* exceeds 99%, and it can effectively inhibit more than 95% of filamentous fungi (Pinder et al., 2025; Shi et al., 2023). In addition to its broad-spectrum antifungal properties, POS exerts a stronger inhibitory effect on the CYP51 enzyme (C-14 demethylase) in the ergosterol synthesis pathway, especially against *Aspergillus* spp. (Pinder et al., 2025; Shi et al., 2023). This advantage may be attributed to the newly added ultra-long hydrophobic side chain on its triazole ring. This specific modification of the chemical structure not only enhances the binding affinity of POS to the CYP51 enzyme but also endows it with stronger cell membrane permeability and membrane-damaging ability. This structural advantage may act synergistically and complementarily with the action of BA: POS-mediated membrane damage creates favorable conditions for BA to enter fungal cells, promoting BA-induced ROS production, thereby amplifying the antifungal effect of POS; meanwhile, the strong hydrophobicity of POS enables it to effectively penetrate the special fungal cell walls rich in melanin or lipids (such as *E. dermatitidis* and *Aspergillus* spp.), laying a structural foundation for the efficient synergy of the BA-POS combination in such fungi with a high risk of drug resistance.

Despite the fact that both ITR and POS are CYP51 enzyme-targeted drugs, there is a significant difference in their synergistic effects, which may be due to the weaker membrane penetration ability of ITR compared to POS. At the same dose, the degree of damage caused by ITR to the fungal cell membrane is limited, making it difficult to form sufficient permeability to promote the efficient entry of BA into cells. As a result, BA cannot mediate the production of adequate ROS, ultimately impairing the synergistic enhancement effect. Furthermore, the regulation of drug efflux pumps and the influence of ROS on efflux pump function are also associated with the synergistic or antagonistic effects produced by the combination of azoles and BA. Studies have shown that after treatment with azole drugs, *Aspergillus* spp. exhibit upregulated transcription of drug efflux transporters (such as Mdr1, AbcA, and Cdr1B) and activation of various stress responses (Pinder et al., 2025), while changes in ROS levels can directly regulate efflux pump activity (Jia and Sun, 2021). The deletion of the mitochondrial protein Bcs1A leads to reduced ROS production and upregulated efflux pump expression, ultimately decreasing the sensitivity of fungi to azole drugs in *A. fumigatus* (Yang et al., 2024); the mutation of the mitochondrial ribosome-binding protein Mba1 enhances fungal resistance to azoles by reducing intracellular ROS content (Zhu et al., 2023). In *C. glabrata*, cells lacking the *GEM1* gene display abnormalities in mitochondrial morphology, increased mitochondrial ROS levels, and significantly upregulated expression of azole drug efflux pumps encoded by the *CDR1* and *CDR2* genes (Okamoto et al., 2023). Notably, only widespread antagonistic effects rather than synergistic interactions were detected for *C. glabrata* in the present study. In contrast to other *Candida* species whose susceptibility variations are mainly modulated by Tac1 and Mrr1, alterations in drug susceptibility of *C. glabrata* are presumably dominated by the transcription factor Pdr1 (Duggan and Usher, 2023; Coste et al., 2004; Morschh User et al., 2007). We hypothesize that BA may specifically boost Pdr1 activity and upregulate ABC transporter genes (*CDR1*, *PDH1*, *SNQ2*, *YOR1*) to accelerate azole efflux (Gale et al., 2023; Vermitsky and Edlind, 2004). Besides, BA–azole combination might disrupt the inherent low azole susceptibility of *C. glabrata*, which could partly explain the antagonistic effect (Vermitsky and Edlind, 2004). Further experimental evidence is required to verify the above speculations.

In contrast, the combination of POS + BA can induce the production of a large amount of ROS, further damaging the integrity of the cell membrane. Moreover, the high binding affinity of POS to the CYP51 enzyme and its ultra-long hydrophobic side chain structure make it difficult to be effluxed by efflux pumps, ensuring effective intracellular drug concentrations and ultimately forming a synergistic effect. Notably, the elevation of ROS may merely represent a cellular stress response triggered by drug treatment, rather than the direct cause of fungal cell death. Additionally, the inherent differences in the regulatory effects of different azole drugs on the fungal transcriptome (including efflux pump genes) further contribute to the higher synergy rate of the BA-POS combination.

Dematiaceous fungi (e.g., *E. dermatitidis*) have special cell walls and poor drug permeability, resulting in low sensitivity to most azoles, and there is no unified treatment strategy for infections caused by them. The isolation rate of multidrug–resistant strains—such as *C. auris* and azole–resistant *Aspergillus* (e.g., *A. fumigatus* carrying the TR34/46 mutation)—has increased year by year. This limits the efficacy of existing treatment regimens and poses a serious challenge to human health.

This study included a large number of clinically isolated strains and confirmed that BA has a stable synergistic antifungal effect with POS. However, the antagonistic effect produced by its combination with ITR should not be ignored. Cancer patients often develop immunosuppression after radiotherapy and chemotherapy, leading to a markedly elevated risk of invasive fungal infections. ITR is a first-line antifungal agent, and co-administration of ITR and BA may occur in these patients. In this study, the antagonism rate of this combination was 25.5%. Accordingly, clinicians should be alert to potential antagonistic interactions during concomitant medication, particularly for *C. glabrata*. In addition, time-kill kinetic assays were not performed in the present study, and thus it cannot be determined whether the combination of BS and POS exerts fungicidal or fungistatic effects. The results of this study are only based on *in vitro* experimental models. Future research may verify the *in vivo* synergistic efficacy of this combination by detecting survival rates, fungal burden in target organs, and histopathological damage. In particular, as a lupane–type pentacyclic triterpenoid derived from natural sources such as birch bark, BA has the advantages of low synthesis cost and low *in vivo* toxicity, making its clinical translation potential significantly superior to that of chemically synthesized potentiators. In recent years, aiming to address the limitations of BA such as poor water solubility and insufficient targeting ability, various drug delivery systems have been gradually developed and applied. For example, BA-containing oleogel formulations for dermatological treatment can improve local delivery efficiency by optimizing the lipophilicity of BA (Smeu et al., 2025); the FA-PBA NPs@Gel hydrogel, constructed using hydroxypropyl chitosan as the carrier, loaded with BA nanoparticles, and modified with folic acid targeting ligands, exhibits core advantages including sustained release, injectability, and enhanced stability in the treatment of osteoarthritis (Liu Q. et al., 2025). Additionally, more advanced delivery systems such as solid lipid NPs, nanostructured lipid carriers, and polymeric micelles are under continuous development (Selepe et al., 2025; Lu et al., 2021; Ghezzi et al., 2021; Viegas et al., 2023). Research advances in the delivery systems provide solid technical support and feasible implementation pathways for the clinical translation of the BA-POS synergistic combination in this study.

## Data Availability

The datasets presented in this study can be found in online repositories. The names of the repository/repositories and accession number(s) can be found at: https://www.ncbi.nlm.nih.gov/, PP069948–PP070390.
