# Supplementary material for: In vitro synergistic activity of betulinic acid combined with azoles against pathogenic fungi
Source: Front Microbiol. 2026 Jun 24;17:1835794. doi: 10.3389/fmicb.2026.1835794 (PMC13341599; doi:10.3389/fmicb.2026.1835794)
Supplement: Supplementary file 1 [file Data_Sheet_1.docx]

###### Table S1 Source of tested strains

| Strains | Isolation site | Underlying disease |
| --- | --- | --- |
| *Aspergillus* spp. |  |  |
| *A. fumigatus* |  |  |
| AF1 | Lung | Invasive aspergillosis |
| AF2 | Lung | Invasive aspergillosis |
| AF3 | Lung | Invasive aspergillosis |
| AF4 | Lung | Invasive aspergillosis |
| AF5 | Lung | Invasive aspergillosis |
| AF6 | Lung | Invasive aspergillosis |
| AF7 | Lung | Invasive aspergillosis |
| AF8 | Lung | Invasive aspergillosis |
| AF9 | Lung | Invasive aspergillosis |
| AF10 | Lung | Invasive aspergillosis |
| AF11 | Lung | Invasive aspergillosis |
| AF12 | External auditory canal | Otomycosis |
| AF13 | External auditory canal | Otomycosis |
| AF14 | Lung | Invasive aspergillosis |
| AF15 | External auditory canal | Otomycosis |
| AF16 | External auditory canal | Otomycosis |
| AF17 | External auditory canal | Otomycosis |
| AF18 | External auditory canal | Otomycosis |
| AF19 | External auditory canal | Otomycosis |
| AF20 | External auditory canal | Otomycosis |
| AF21 | External auditory canal | Otomycosis |
| AF22 | External auditory canal | Otomycosis |
| AF23 | External auditory canal | Otomycosis |
| AF24 | External auditory canal | Otomycosis |
| AF25 | External auditory canal | Otomycosis |
| AF26 | External auditory canal | Otomycosis |
| AF27 | External auditory canal | Otomycosis |
| *A. flavus* |  |  |
| AL1 | External auditory canal | Otomycosis |
| AL2 | External auditory canal | Otomycosis |
| AL3 | External auditory canal | Otomycosis |
| AL4 | External auditory canal | Otomycosis |
| AL5 | External auditory canal | Otomycosis |
| AL6 | External auditory canal | Otomycosis |
| AL7 | External auditory canal | Otomycosis |
| AL8 | External auditory canal | Otomycosis |
| AL9 | Lung | Invasive aspergillosis |
| AL10 | Lung | Invasive aspergillosis |
| NRRL 3357 | Peanut cotyledons | Not documented |
| ATCC 204304 | Not documented | Not documented |
| *A. terreus* |  |  |
| AT1 | External auditory canal | Otomycosis |
| AT2 | External auditory canal | Otomycosis |
| AT3 | External auditory canal | Otomycosis |
| AT4 | External auditory canal | Otomycosis |
| AT5 | External auditory canal | Otomycosis |
| AT6 | Lung | Invasive aspergillosis |
| AT7 | Lung | Invasive aspergillosis |
| AT8 | Lung | Invasive aspergillosis |
| *A. niger* |  |  |
| AN1 | External auditory canal | Otomycosis |
| AN2 | External auditory canal | Otomycosis |
| AN3 | External auditory canal | Otomycosis |
| AN4 | External auditory canal | Otomycosis |
| AN5 | Lung | Invasive aspergillosis |
| *Cryptococcus neoformans* |  |  |
| Y1 | Lung | Cryptococcal pneumonia |
| Y2 | Lung | Cryptococcal pneumonia |
| Y3 | Lung | Cryptococcal pneumonia |
| Y6 | Brain | Cryptococcal meningitis |
| Y7 | Brain | Cryptococcal meningitis |
| Y8 | Brain | Cryptococcal meningitis |
| Y9 | Brain | Cryptococcal meningitis |
| Y11 | Brain | Cryptococcal meningitis |
| Y12 | Brain | Cryptococcal meningitis |
| *Exophiala dermatitidis* |  |  |
| BMU00028 | Brain | Phaeohyphomycosis |
| BMU00029 | Skin | Phaeohyphomycosis |
| BMU00030 | Skin | Phaeohyphomycosis |
| BMU00031 | Skin | Phaeohyphomycosis |
| BMU00034 | Skin | Phaeohyphomycosis |
| BMU00035 | Skin | Phaeohyphomycosis |
| BMU00036 | Skin | Phaeohyphomycosis |
| BMU00037 | Skin | Phaeohyphomycosis |
| BMU00038 | Skin | Phaeohyphomycosis |
| BMU00039 | Skin | Phaeohyphomycosis |
| BMU00040 | Skin | Phaeohyphomycosis |
| BMU00041 | Skin | Phaeohyphomycosis |
| 109140 | Skin | Phaeohyphomycosis |
| 109144 | Skin | Phaeohyphomycosis |
| 109145 | Skin | Phaeohyphomycosis |
| 109148 | Skin | Phaeohyphomycosis |
| 109152 | Skin | Phaeohyphomycosis |
| NPRC 3.8.653 | Skin | Phaeohyphomycosis |
| NPRC 3.8.654 | Skin | Phaeohyphomycosis |
| NPRC 3.8.655 | Skin | Phaeohyphomycosis |
| NPRC 3.8.656 | Skin | Phaeohyphomycosis |
| *Candida* spp. |  |  |
| *C. auris* |  |  |
| 381 | External auditory canal | Otomycosis |
| 382 | Skin | Burn wound infection |
| 383 | Blood | Candidaemia |
| 384 | Blood | Candidaemia |
| 385 | Blood | Candidaemia |
| 386 | Blood | Candidaemia |
| 387 | Blood | Candidaemia |
| 388 | Blood | Candidaemia |
| 389 | BAL/lung | Pneumonia |
| 390 | Blood | Candidaemia |
| *C. albicans* |  |  |
| R8 | Vagina | Candidal vaginitis |
| R9 | Vagina | Candidal vaginitis |
| R14 | Oral cavit | Mycotic stomatitis |
| R66 | Blood | Candidaemia |
| 122473 | Blood | Candidaemia |
| 129968 | Blood | Candidaemia |
| 122843 | Vagina | Candidal vaginitis |
| 123118 | Blood | Candidaemia |
| 130110 | Blood | Candidaemia |
| *C. glabrata* |  |  |
| 122512 | Blood | Candidaemia |
| 122889 | Blood | Candidaemia |
| 122127 | Vagina | Candidal vaginitis |
| 130133 | Blood | Candidaemia |
| 5448 | Blood | Candidaemia |
| *C. tropicalis* |  |  |
| 122115 | Blood | Candidaemia |
| 122543 | Vagina | Candidal vaginitis |
| *C. parapsilosis* |  |  |
| 117406 | Blood | Candidaemia |
| 22019 | Blood | Candidaemia |

###### Table S2 Primer sets and corresponding amplification targets

| Target gene | Primer | Primer DNA sequence (5’–3’) |
| --- | --- | --- |
| ITS | ITS1 | TCCGTAGGTGAACCTGCGG |
|  | ITS4 | TCCTCCGCTTATTGATATGC |
| calmodulin | cmd5 | CCGAGTACAAGGAGGCCTTC |
|  | cmd6 | CCGATAGAGGTCATAACGTGG |
| beta–tubulin | Bt2a | GGTAACCAAATCGGTGCTGCTTTC |
|  | Bt2b | ACCCTCAGTGTAGTGACCCTTGGC |


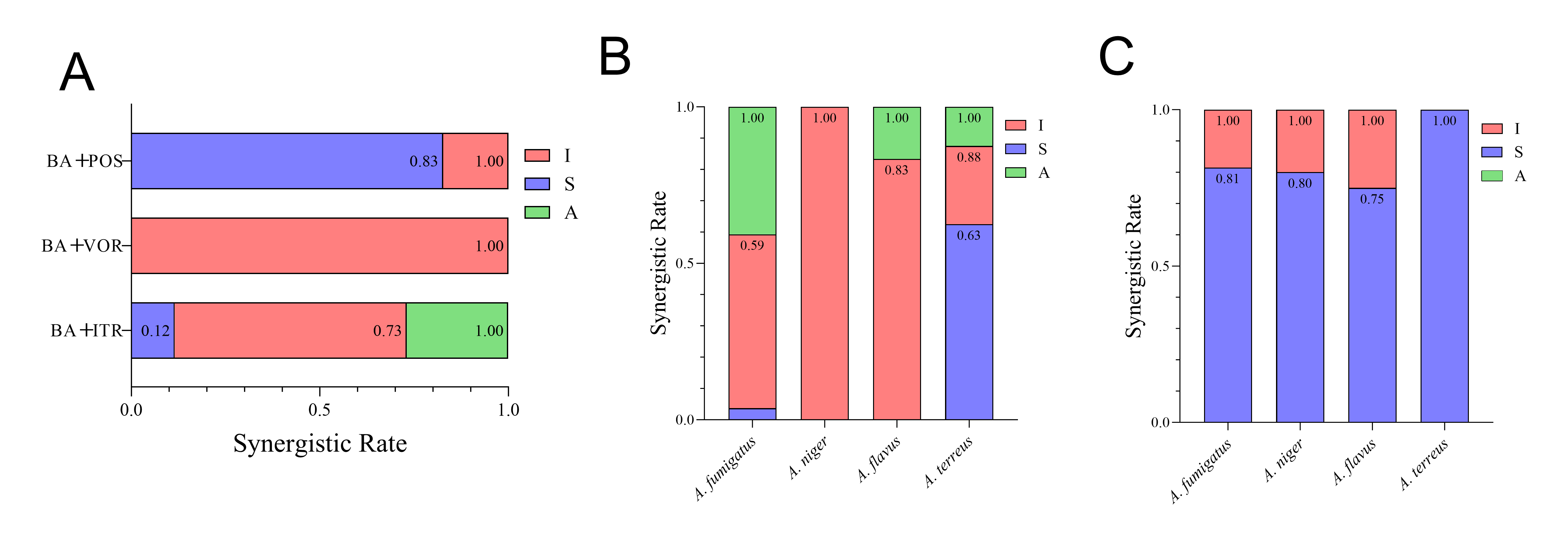


###### Fig. S1 The Synergistic Rate of BA Combined with Azoles against *Aspergillus* spp.

Note: A, Interaction profile of BA combined with ITR, VOR and POS against *Aspergillus* spp.; B, BA/ITR interaction profiles across *Aspergillus* species; C, BA/POS interaction profiles across *Aspergillus* species. S: synergy (FICI≤0.5); I: indifference (FICI from >0.5 to ≤4); A, antagonism (FICI of >4). BA, betulinic acid; ITR, itraconazole; VOR, voriconazole; POS, posaconazole. The synergism rate was calculated by dividing the number of strains exhibiting synergism by the total number of strains tested.


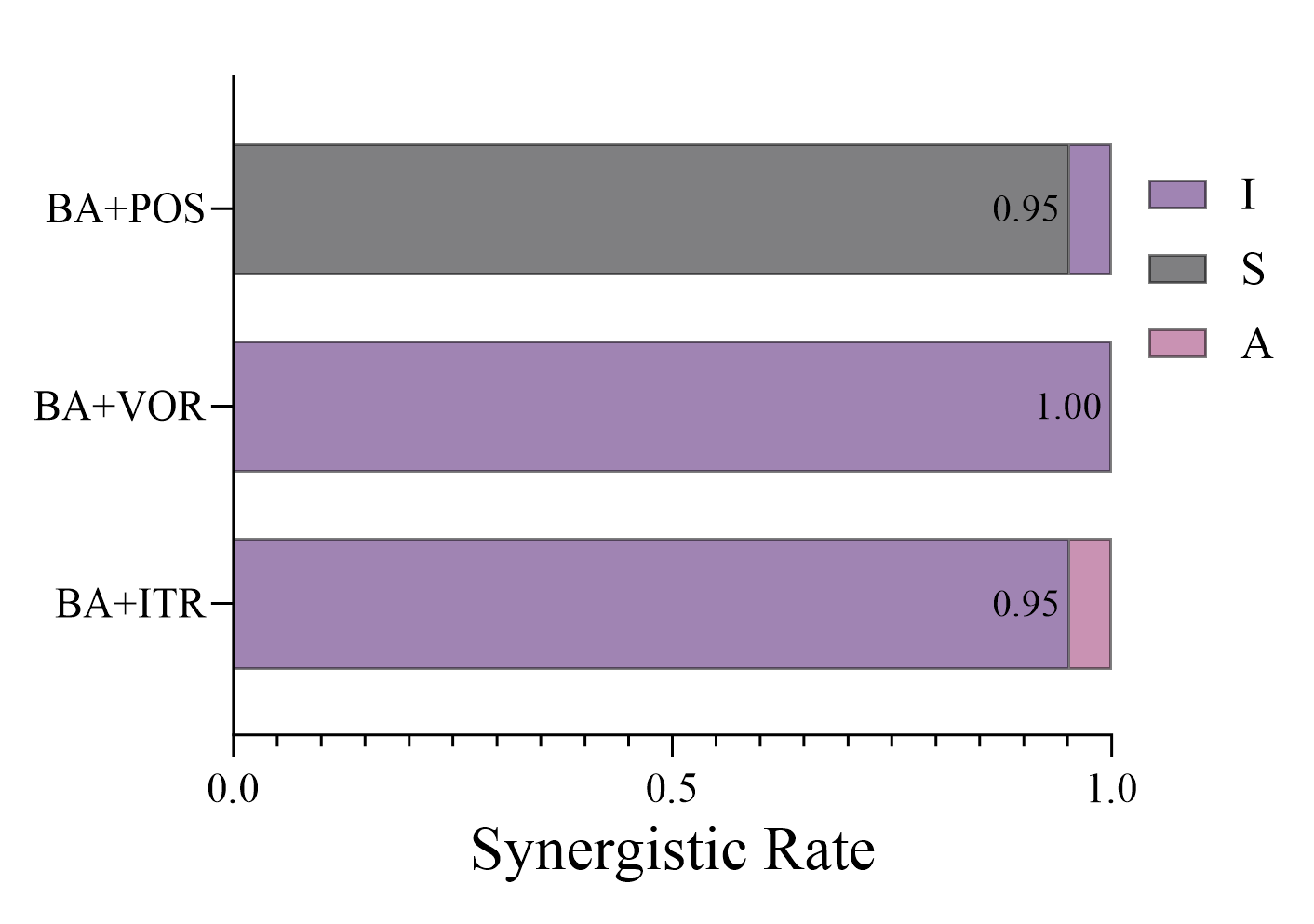


###### Fig. S2 The Synergistic Rate of BA Combined with Azoles against *E. dermatitidis*

Note: ITR, itraconazole; VOR, voriconazole; POS, posaconazole; BA, betulinic acid; S, synergy (FICI ≤0.5); I, indifference (no interaction, FICI from >0.5 to ≤4); A, antagonism (FICI of >4). The synergism rate was calculated by dividing the number of strains exhibiting synergism by the total number of strains tested.

######
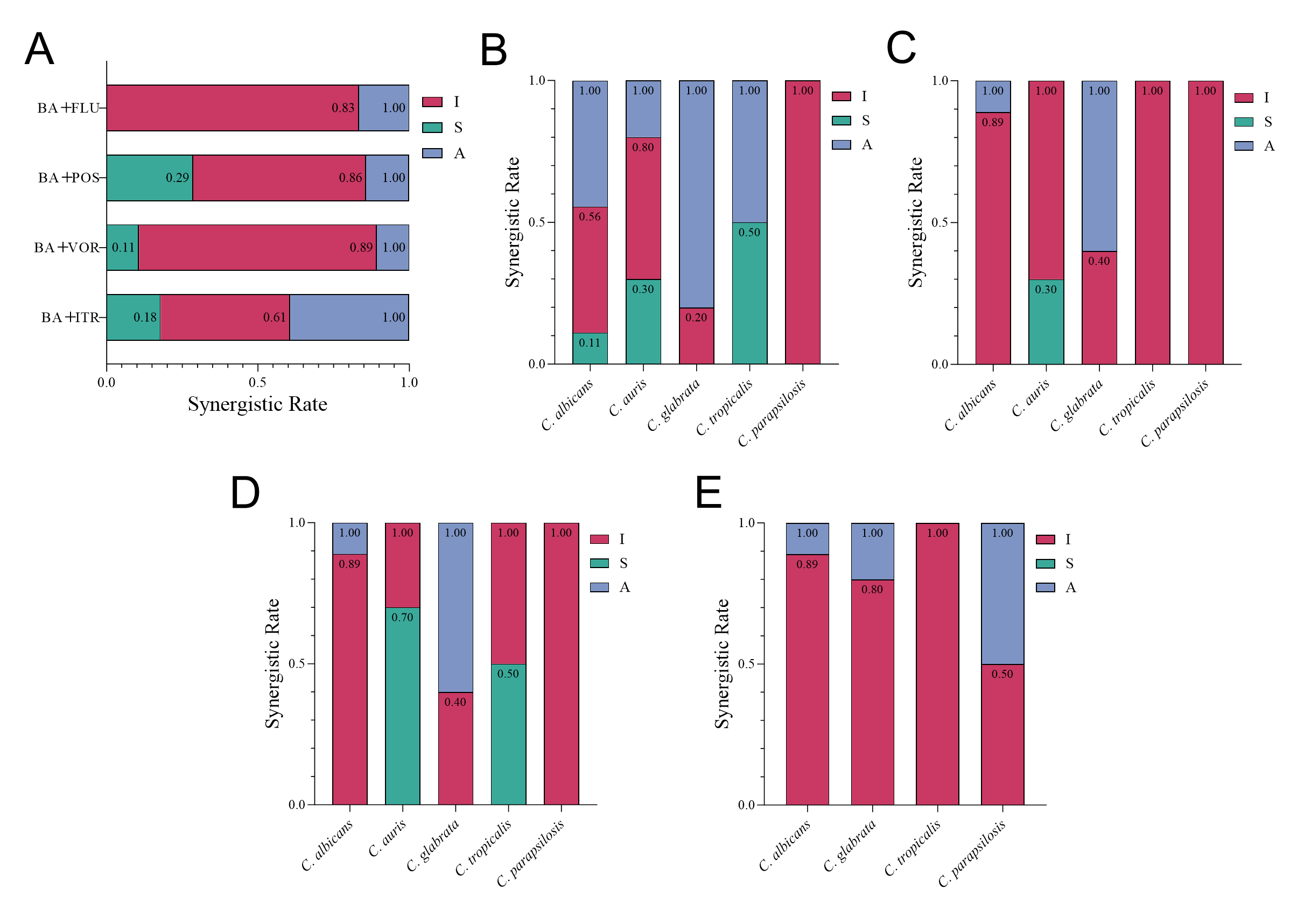
**Fig. S3 The Synergistic Rate of BA Combined with Azoles against *Candida* spp.**

Note: A, interaction profile of BA in combination with azoles against *Candida* spp.; B, BA/ITR interaction profiles across *Candida* species; C, BA/VOR interaction profiles across *Candida* species; D, BA/POS interaction profiles across *Candida* species; E, BA/FLC interaction profiles across *Candida* species. S: synergy (FICI≤0.5); I: indifference (FICI from >0.5 to ≤4); A, antagonism (FICI of >4); BA, betulinic acid; ITR: itraconazole; VOR, voriconazole; POS: posaconazole; FLC, fluconazole. The synergism rate was calculated by dividing the number of strains exhibiting synergism by the total number of strains tested.


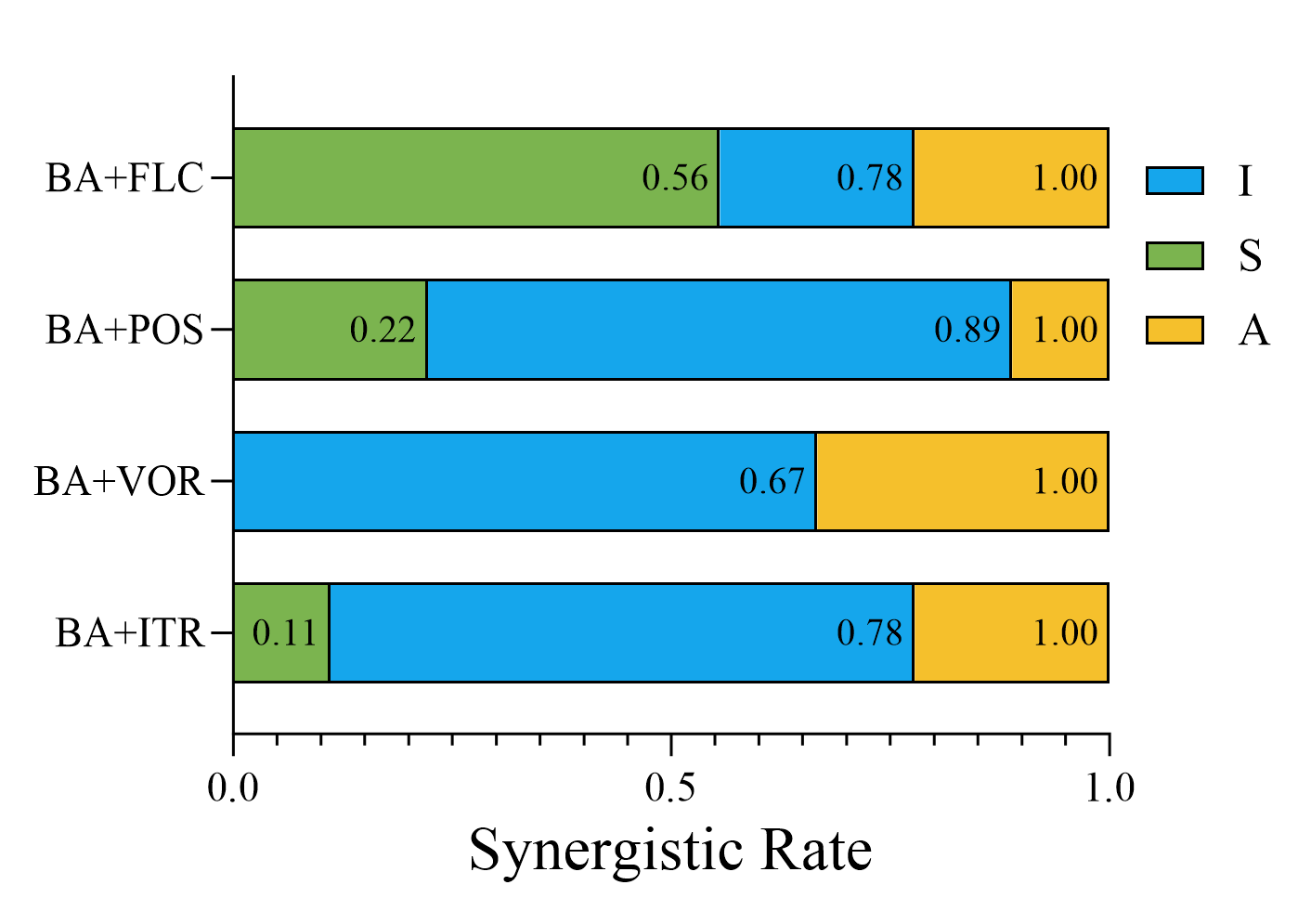


###### Fig. S4 The Synergistic Rate of BA Combined with Azoles against *C. neoformans*

Note: S: synergy (FICI≤0.5); I: indifference (FICI from >0.5 to ≤4); A, antagonism (FICI of >4); BA, betulinic acid; ITR: itraconazole; VOR: voriconazole; POS: posaconazole; FLC, fluconazole. The synergism rate was calculated by dividing the number of strains exhibiting synergism by the total number of strains tested.


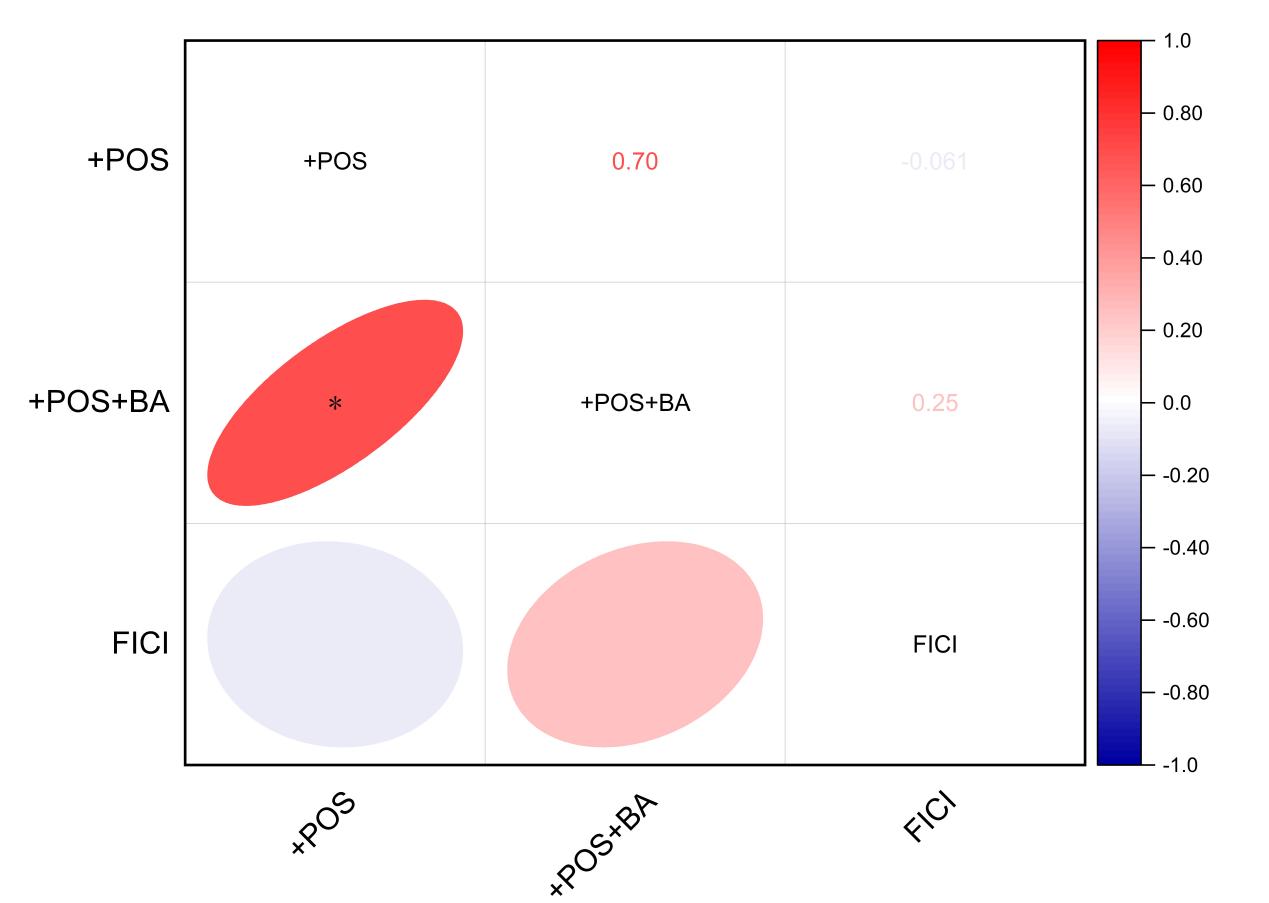


###### Fig. S5 Correlation analysis of ROS levels and synergistic activity

Note: The heatmap illustrates Spearman correlation coefficients (r) between the fractional inhibitory concentration index (FICI) and intracellular ROS levels induced by posaconazole alone (+POS) or in combination with betulinic acid (+POS+BA). The color gradient (red to blue) represents positive to negative correlation strength. ROS levels induced by +POS and +POS+BA showed a significantly strong positive correlation (r = 0.70, *P* < 0.05). No statistically significant correlation was detected between FICI values and +POS+BA-triggered ROS levels (r = 0.25, *P* > 0.05), nor between FICI values and +POS-triggered ROS levels (r = -0.06, *P* > 0.05).
